# Emergence of Carbapenem- and Tigecycline-Resistant *Proteus cibarius* of Animal Origin

**DOI:** 10.3389/fmicb.2020.01940

**Published:** 2020-08-14

**Authors:** Yan Li, Qian Wang, Kai Peng, Yuan Liu, Ruichao Li, Zhiqiang Wang

**Affiliations:** ^1^College of Veterinary Medicine, Yangzhou University, Yangzhou, China; ^2^Institute of Comparative Medicine, Yangzhou University, Yangzhou, China; ^3^Jiangsu Co-innovation Center for Prevention and Control of Important Animal Infectious Diseases and Zoonoses, Yangzhou University, Yangzhou, China

**Keywords:** tigecycline resistance, carbapenem resistance, coexistence, plasmid, chromosome

## Abstract

The emergence of *tet*(X) and carbapenemase genes in Enterobacterales pose significant challenges to the treatment of infectious diseases. Convergence of these two categories of genes in an individual pathogen would deteriorate the antimicrobial resistance (AMR) crisis furthermore. Here, tigecycline-resistant Enterobacterales strains were isolated and detected with carbapenemase genes, characterized by antimicrobial susceptibility testing, PCR, conjugation assay, whole genome sequencing, and bioinformatics analysis. Three tigecycline-resistant isolates consisting of one plasmid-mediated *tet*(X4)-bearing *Escherichia fergusonii* and two chromosomal *tet*(X6)-bearing *Proteus cibarius* were recovered from chicken feces. The *tet*(X4) was located on a conjugative IncX1 plasmid pHNCF11W-tetX4 encoding the identical structure as reported *tet*(X4)-bearing IncX1 plasmids in *Escherichia coli*. Among two *P. cibarius* strains, *tet*(X6) was located on two similar chromosomal MDR regions with genetic contexts IS*26*-*aac(3)-IVa*-*aph(4)-Ia*-IS*Ec59*-*tnpA*-*tet*(X6)-*orf*-*orf*-IS*CR2*-*virD2*-*floR*-IS*CR2*-*glmM*-*sul2* and IS*26*-*aac(3)-IVa*-*aph(4)-Ia*-IS*Ec59*-*tnpA*-*tet*(X6)-*orf*-*orf*-IS*CR2*-*glmM*-*sul2*. Apart from *tet*(X6), *P. cibarius* HNCF44W harbored a novel transposon Tn*6450b* positive for *bla*_NDM–__1_ on a conjugative plasmid. This study probed the genomic basis of three *tet*(X)-bearing, tigecycline-resistant strains, one of which coharbored *bla*_NDM–__1_ and *tet*(X6), and identified *P. cibarius* as the important reservoir of *tet*(X6) variants. Emergence of *P. cibarius* encoding both *bla*_NDM–__1_ and *tet*(X6) reveals a potential public health risk.

## Introduction

Antimicrobial resistance (AMR) is a serious threat to public health globally. Carbapenem and tigecycline are regarded as vital antimicrobials reserved for clinical use due to their broad antibacterial spectrum. However, the increasing spread of carbapenemase-encoding genes has rendered carbapenem-resistant Enterobacteriaceae (CRE) infection a great threat (Du et al., 2018; Tacconelli et al., 2018). Plasmid-mediated resistance genes *tet*(X3) and *tet*(X4) conferring high-level resistance to tigecycline in Enterobacterales and *Acinetobacter* has been found to be ubiquitous in animals and food of animal origin (Bai et al., 2019; He et al., 2019; Sun et al., 2019; Li et al., 2020c). This undermines the efficacy of tigecycline as the last-resort drug in treating MDR bacterial infections, especially those caused by carbapenem-resistant, Gram-negative bacteria, such as CRE.

Initially, *tet*(X) genes were mainly found in *Bacteroides* species and bacteria derived from environmental microbiota (Forsberg et al., 2015; Tian et al., 2019). Alarmingly, novel *tet*(X) variants are being identified from the pathogens of animal, food, and human origins (Li et al., 2019; Wang et al., 2019; He D. et al., 2020; Liu et al., 2020; Peng et al., 2020), which implies that *tet*(X) is expanding from commensals to pathogens (Aminov, 2013). Thus, the convergence of mobile tigecycline-resistant *tet*(X) and carbapenem-resistant genes in Enterobacterales may be inevitable. *Acinetobacter* strains coharboring *tet*(X) and *bla*_NDM–__1_ were identified from samples of waterfowl and dairy cows recently (Cui et al., 2020; He T. et al., 2020), highlighting the potential convergence risk of both critical resistance genes among bacteria. However, Enterobacterales carrying both *tet*(X) and *bla*_NDM–__1_ is unknown, which constitutes a more severe concern. To cover this knowledge gap, we performed a tigecycline-resistant Enterobacterales screening pilot program and identified a *Proteus cibarius* coharboring both *tet*(X6) and *bla*_NDM–__1_, implying that wide surveillance of Enterobacterales conferring resistance to carbapenems and tigecycline among bacteria should be performed worldwide.

## Materials and Methods

### Bacterial Isolates

A total of 16 chicken fecal samples were collected from four chicken farms in Henan province, China, in 2019. Tigecycline-resistant Enterobacterales were selected on MacConkey agar plates containing tigecycline (4 mg/L). The species of purified tigecycline-resistant isolates was identified by 16S rRNA gene sequencing and further confirmed by analyzing WGS data. The *tet*(X) and carbapenem-resistant genes were determined by PCR with reported primers (Dallenne et al., 2010; He et al., 2019).

### Antimicrobial Susceptibility Testing

The antimicrobial susceptibility testing (AST) of *tet*(X)-positive tigecycline-resistant strains against 13 antibiotics was performed based on the broth microdilution method with *Escherichia coli* ATCC 25922 as the quality control and interpreted according to CLSI guidelines (CLSI, 2018). The tigecycline non-susceptibility in Enterobacterales was interpreted with an MIC of ≥4 mg/L (Marchaim et al., 2014).

### Conjugation Assay and S1-PFGE

A conjugation assay was conducted using rifampicin-resistant *E. coli* C600 (Rif^r^) as the recipient to investigate the transferability of *tet*(X) and *bla*_NDM–__1_. The donor and recipient strains were mixed at a ratio of 1:4 on a filter, followed by culturing on LB agar plates at 30 and 37°C, respectively, overnight and screening on MacConkey plates containing tigecycline (4 mg/L) or meropenem (2 mg/L) with rifampicin (300 mg/L). Transconjugants were confirmed with PCR. S1 nuclease pulsed-field gel electrophoresis (S1-PFGE) was utilized to characterize the plasmid profiles.

### Whole Genome Sequencing and Bioinformatics Analysis

Genomid DNA (gDNA) was purified with the TIANamp Genomic DNA Kit and quantified by Qubit 4 Fluorometer. Short-read Illumina sequencing and long-read Nanopore sequencing were combined to generate complete genome sequences (Li et al., 2018). The *de novo* assembled sequences were annotated by RAST^1^. Antibiotic resistance genes (ARGs) and plasmid replicon types were determined using the ResFinder and PlasmidFinder^2^. BRIG and Easyfig were used to compare the plasmid and MDR structures (Alikhan et al., 2011; Sullivan et al., 2011).

### Functional Confirmation of *tet*(X6) and Phylogenetic Analysis

To confirm the resistance function of the identified *tet*(X6) variant, TA-cloning and AST were performed. Briefly, the new variant, together with its natural promoter sequence, was amplified by PCR using primers HNCF44W-F/AGCGAACAAGAATATGACTTTACT and HNCF44W-R/CGCCTTTCTGTTTTATAGATTCAT, cloned into a pMD19-T vector and transformed chemically into *E. coli* DH5α. The resistance phenotype of *tet*(X6) was tested by measuring the MICs of different tetracycline antibiotics. Phylogenetic analysis of Tet(X) amino acid sequences were conducted in MEGA X using the neighbor-joining method (Kumar et al., 2018).

### Nucleotide Sequence Accession Numbers

The complete sequences obtained in this study have been deposited in the GenBank database under BioProject number PRJNA625637. The novel *bla*_NDM–__1_-bearing Tn*6450b* was also deposited in the NCBI database (MT701524).

## Results and Discussion

### Identification of Tigecycline-Resistant Enterobacterales

Three tigecycline-resistant, *tet*(X)-positive Enterobacterales strains were isolated from 16 samples (18.8%), which consisted of *Escherichia fergusonii* HNCF11W carrying *tet*(X4), *P. cibarius* HNCF43W, and HNCF44W positive for a *tet*(X6) variant. Notably, HNCF44W also carried the *bla*_NDM–__1_ gene. Antimicrobial susceptibility testing revealed that the three tigecycline-resistant strains were resistant to multiple antibiotics, including kanamycin, florfenicol, amoxicillin, and tetracyclines, showing high-level resistance to tigecycline (Supplementary Table S1). In addition, the NDM-producing strain HNCF44W was also resistant to meropenem (>16 mg/L). S1-PFGE indicated that two plasmids of ca. 130 and 55 kb were observed in *tet*(X4)-bearing *E. fergusonii* HNCF11W, and one plasmid of ca. 140 kb existed in *tet*(X6)-bearing *P. cibarius* HNCF44W, but no plasmid existed in *P. cibarius* HNCF43W (Supplementary Figure S1). To uncover the genetic locations of *tet*(X) and *bla*_NDM–__1_, complete genome sequences of the three strains were finished successfully.

### Genome Characterization of Tigecycline-Resistant *E. fergusonii*

The *tet*(X4)-bearing *E. fergusonii* harbored one chromosome (4,584,794 bp, ST10543); one p0111-like plasmid pHNCF11W-130 kb (130,643 bp) harboring *aadA1*, *floR*, *tet*(A), *dfrA14*, *bla*_TEM–__209_, *bla*_OXA–__10_, *cmlA1*, *arr-3*, and *qnrS1*; and one typical IncX1 plasmid pHNCF11W-tetX4 (57,105 bp) encoding *tet*(X4) in the genetic structure of IS*26*-*abh*-*tet*(X4)-IS*CR2*-*virD2*-*floR-*△IS*CR2* coupled with *aadA2*, *lnu(F)*, *tet*(A), and *bla*_SHV–__12_. A BLASTn search of pHNCF11W-tetX4 against the nr database retrieved other *tet*(X4)-bearing plasmids p1916D18-1 (59,353 bp), p1916D6-2 (59,351 bp), and pYY76-1-2 (57,104 bp) of swine and cattle origins, sharing nearly identical sequences (Supplementary Figure S2). Both pHNCF11W-tetX4 and pHNCF11W-130 kb were transferred into the recipient strain, demonstrating that IncX1-type *tet*(X4)-bearing plasmids were mobilizable and spread in Enterobacterales of animal origin (Chen et al., 2019). Although *tet*(X4) was initially found in plasmids of IncFIB and IncQ1 types (He et al., 2019; Sun et al., 2019), IncX1 plasmids are common in various Enterobacterales and associated with various resistance genes (Dobiasova and Dolejska, 2016; Li et al., 2020b), which potentially increases the transmission of *tet*(X4) between different Enterobacterales.

### Characterization of *Proteus cibarius* Coharboring *tet*(X6) and *bla*_NDM–__1_

**FIGURE 1:**
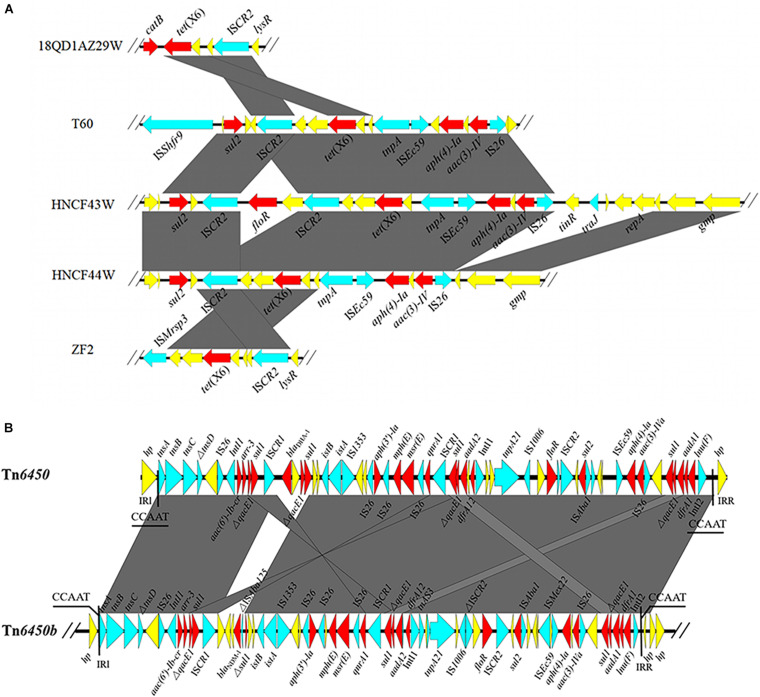
**(A)** Genetic environments of *tet*(X6) from different strains. **(B)** Linear alignment of *bla*_NDM–__1_-bearing Tn*6450b* in pHNCF44W_NDM-1 and reported Tn*6450*. The red arrows denote the resistance genes; the blue arrows represent the mobile elements; the yellow arrows signify other CDSs. The shadow parallelograms stand for homologous genetic regions.

HNCF43W harbored a 3,965,977 bp chromosome. HNCF44W contained a 3,929,176 bp chromosome and one *bla*_NDM–__1_-bearing plasmid pHNCF44W_NDM-1 of 139,490 bp in size. A conjugation assay failed to recover transconjugants positive for *tet*(X6). The *tet*(X6) gene was located on large MDR regions (IS*26*-*aac(3)-IVa*-*aph(4)-Ia*-IS*Ec59*-*tnpA*-*tet*(X6)-*orf*-*orf*-IS*CR2*-*virD2*-*floR*-IS*CR2*-*glmM*-*sul2* and IS*26*-*aac(3)-IVa*-*aph(4)-Ia*-IS*Ec59*-*tnpA*-*tet*(X6)-*orf*-*orf*-IS*CR2*-*glmM*-*sul2*, respectively) embedded in chromosomes of both *P. cibarius* strains (Figure 1A), and the MDR regions may derive from recombination of *tet*(X6)-bearing elements and reported plasmids p19110F47-2 (CP046044) and pJXP9 (MK673549) (Supplementary Figure S3). The two *tet*(X6)-bearing MDR regions differed by *virD2*-*floR*-IS*CR2*, which was a potential mobile segment (Doublet et al., 2005). IS*CR2* is the major mobile element associated with *tet*(X3), *tet*(X4), and *tet*(X5) (Bai et al., 2019; He et al., 2019; Sun et al., 2019; Li et al., 2020a). Recently, three reports demonstrated *tet*(X6) could be embedded in ICEs or other MDR regions, all of which harbor IS*CR2* (He D. et al., 2020; Liu et al., 2020; Peng et al., 2020), suggesting it may facilitate the transfer of *tet*(X6) to other plasmids or chromosomes.

**FIGURE 2:**
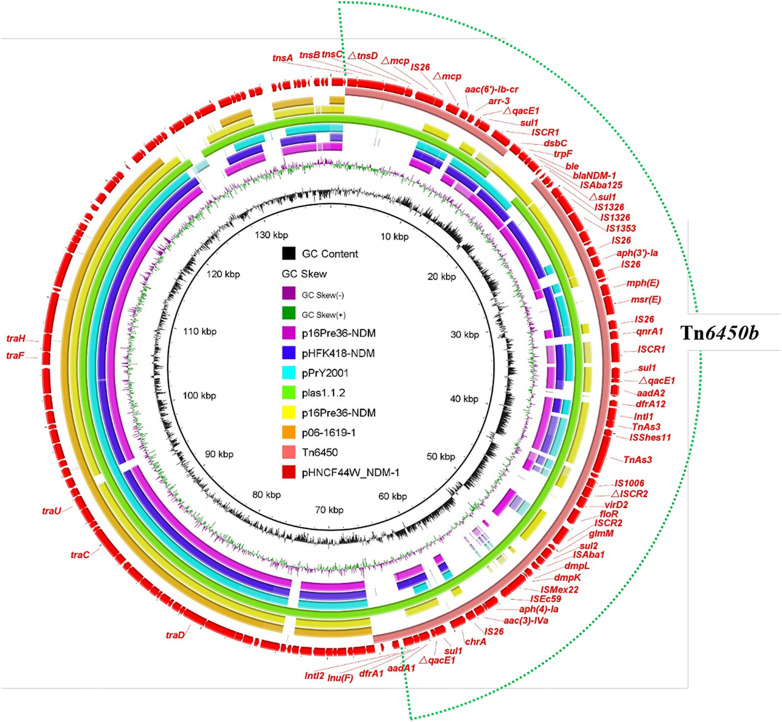
Alignment of the plasmid pHNCF44W_NDM-1 identified in this study with other similar plasmids. The outermost red circle denotes the reference plasmid pHNCF44W_NDM-1. The location of Tn*6450b* is highlighted in the dotted circle.

The *bla*_NDM–__1_ gene in HNCF44W was located on an MDR plasmid pHNCF44W_NDM-1 carrying resistance genes *aac(6′)-Ib, arr-3, qacE1, sul1, sul2, aph(3′)-la, mph(E), msr(E), qnrA1, aadA1, aadA2, dfrA1, dfrA12, floR, aac(3)-IVa*, and *lnu(F)*. No plasmid replicon was identified, but pHNCF44W_NDM-1 was very similar to plasmid plas1.1.2 (CP047114) from *Proteus mirabilis* of swine origin; it differed mainly in the *bla*_NDM–__1_-bearing region (Figure 2). The *bla*_NDM–__1_ gene and other resistance genes were found in a 65-kb MDR region, showing high similarity to transposon Tn*6450* but with *bla*_DHA–__1_ replaced by *bla*_NDM–__1_ (Chen et al., 2018), designated as Tn*6450b* here (Figure 1B). Other similar plasmids or transposons were found in the nr database, including p16Pre36-NDM from *Providencia rettgeri*, pHFK418-NDM from *P. mirabilis*, pPrY2001 from *P. rettgeri*, p16Pre36-NDM from *P. rettgeri*, p06-1619-1 from *P. rettgeri*, and Tn*6450* from *P. mirabilis*. The plasmid pHNCF44W_NDM-1 carrying *bla*_NDM–__1_ was successfully transferred into *E. coli* EC600 at 30°C. Notably, IS*CR2*-*lysR*-*floR*-*virD2*-△IS*CR2* structure in pHNCF44W_NDM-1 was also found in a *tet*(X6)-bearing chromosomal segment, rendering this a Tn*6450b*-bearing plasmid with the potential ability to capture *tet*(X6) under certain circumstances, which warrants further surveillance.

### Functional Analysis of *tet*(X6) and Its Evolution

**FIGURE 3:**
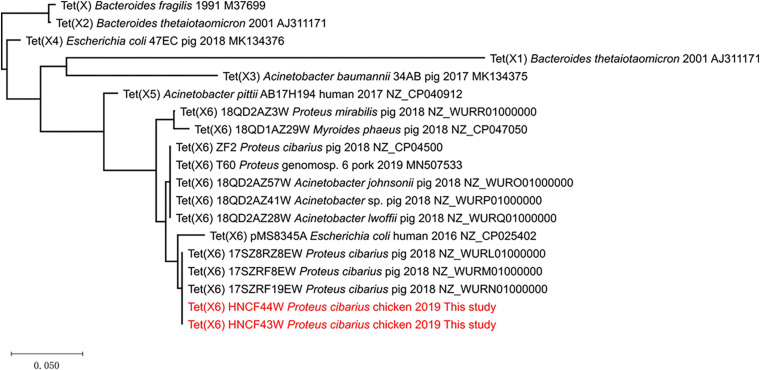
The phylogenetic tree of Tet(X) variants. The tree is drawn to scale with branch lengths in the same units as those of the evolutionary distances used to infer the phylogenetic tree. The evolutionary distances were computed using MEGA X and are in the units of the number of amino acid substitutions per site.

Recently, several reports have characterized different *tet*(X6) variants from diverse bacteria (He D. et al., 2020; Liu et al., 2020; Peng et al., 2020; Xu et al., 2020). The *tet*(X6) variant carried by HNCF44W and HNCF43W is the same as that reported in *P. cibarius* from swine (Liu et al., 2020). The expression of *tet*(X6) in the cloning vector pMD19-T-tet(X6) in *E. coli* DH5α conferred resistance to tetracycline (64 mg/L) and tigecycline (16 mg/L) with 32-fold increments compared to that of DH5α-pMD19-T, indicating that this *tet*(X6) variant can confer tigecycline resistance (Supplementary Table S2). The 1137 bp *tet*(X6) variant encoded 378 amino acids and showed 84.7, 66.3, 84.9, 79.9, 87.8, 93.7, and 98.7% amino acid sequence identity to Tet(X), Tet(X1), Tet(X2), Tet(X3), Tet(X4), Tet(X5), and Tet(X6), respectively (Figure 3) with 5, 8, and 16 amino acid substitutions to Tet(X6) (MN507533, T60), Tet(X6) (CP025402, pMS8345A), and Tet(X6) (NZ_CP047050, 18QD1AZ29W).

Phylogenetic analysis of Tet(X) indicated that of all reported Tet(X6) variants clustered in a clade, most related to Tet(X5) (Figure 3). Although most *tet*(X6) variants were found in *Proteus* and *Acinetobacter* spp., analysis of the NCBI database shows that *E. coli* positive for *tet*(X6) existed as early as 2003 in Denmark (JWJM01000174), indicating the transmission of *tet*(X6) occurred previously. Furthermore, chromosomal *tet*(X6)-bearing ICEs and MDRs are found in *Proteus*, *Acinetobacter*, and *Myroides phaeus* (He D. et al., 2020; Liu et al., 2020; Peng et al., 2020). Plasmid-mediated *tet*(X6) in *Proteus*, *Acinetobacter*, and *E. coli* also emerged according to published reports (Cui et al., 2020; Li et al., 2020c; Xu et al., 2020), implying *tet*(X6) is at the stage of being expanded via different mobile elements. This highlights that *tet*(X6) variants would be the next possible prominent *tet*(X) allele, approaching the widespread situation of *tet*(X4) in *E. coli* (Bai et al., 2019; He et al., 2019; Sun et al., 2019). Nomenclature of *tet*(X) by different allele numbers is not recommended because many of the alleles are 85–100% aa identical^3^, but an identical *tet*(X) naming system may not benefit research communications and AMR surveillance, especially as different *tet*(X) alleles possess different resistance phenotypes, genetic contexts, hosts, and evolutionary routes as more research reveals. For example, *tet*(X4) was found to be more common in *E. coli* than *tet*(X6) owing to the fact that *tet*(X4) has merged into conjugative plasmids adapted in *E. coli*. Also, the plasmids coharboring both *bla*_NDM–__1_ and *tet*(X) in *Acinetobacter* spp. (Cui et al., 2020) could be divided into *tet*(X3)- or *tet*(X6)-bearing plasmids, which could make it difficult to decipher the accurate different genetic contexts if only *tet*(X) represented the two alleles simultaneously. To better investigate the molecular epidemiology, genomic evolution, and functional analysis and avoid confusion in *tet*(X) designations, a clear nomenclature scheme acceptable to researchers worldwide should be proposed as soon as possible. Because *mcr* genes exist in certain bacterial species as housekeeping genes and certain alleles leap over species boundary limits to Enterobacterales via mobile elements like Tn*6330* (Li et al., 2016; Shen et al., 2020), *tet*(X) genes may behave similarly because they exist as core genes in certain bacteria and transfer to Enterobacterales by mobile elements like IS*CR2*. Thus, the *mcr* nomenclature scheme may be a good example when it comes to *tet*(X) designation (Partridge et al., 2018). More research should be performed to put forward a widely accepted *tet*(X) allele numbering system to facilitate research communications.

## Conclusion

To conclude, we characterized three *tet*(X)-bearing Enterobacterales strains of chicken origin and found that one *P. cibarius* coharbored both *bla*_NDM–__1_ and *tet*(X6). To the best of our knowledge, this is the first report of convergence of *tet*(X6) and *bla*_NDM–__1_ genes in Enterobacterales, highlighting the importance of continuous surveillance of tigecycline- and carbapenem-resistant Enterobacterales of animal origin, which may act as the potential reservoir of clinical Enterobacterales resistant to tigecycline and carbapenems.

## Data Availability Statement

The datasets presented in this study can be found in online repositories. The names of the repository/repositories and accession number(s) can be found in the article/Supplementary Material.

## Author Contributions

RL and ZW: conceptualization, writing – review and editing, and supervision. YaL and RL: methodology. YaL and QW: investigation. QW, KP, and YuL: data curation and visualization. YaL: writing – original draft preparation. All authors have read and agreed to the published version of the manuscript.

## Conflict of Interest

The authors declare that the research was conducted in the absence of any commercial or financial relationships that could be construed as a potential conflict of interest.

## Footnotes

1 https://rast.nmpdr.org/

2 http://www.genomicepidemiology.org/

3 http://faculty.washington.edu/marilynr/
