# Supplementary material for: Emergence of Carbapenem- and Tigecycline-Resistant Proteus cibarius of Animal Origin
Source: Front Microbiol. 2020 Aug 14;11:1940. doi: 10.3389/fmicb.2020.01940 (PMC7457074; doi:10.3389/fmicb.2020.01940)
Supplement: Supplementary file 1 [file Data_Sheet_1.docx]

**Supplementary Data**

**Table S1. Antimicrobial susceptibility testing (MICs, mg/L) of the strains against different antimicrobials.**

| IDs^a^ | Species | Sources | Antimicrobials | | | | | | | | | | | | |
| --- | --- | --- | --- | --- | --- | --- | --- | --- | --- | --- | --- | --- | --- | --- | --- |
|  |  |  | MEM^a^ | DOX | AMX | FFC | KAN | CL | CIP | TIG | OXY | TET | MIN | CFF | ENR |
| HNCF43W | *Proteus cibarius* | Henan | 2 | 64 | >64 | >64 | 32 | >256 | 128 | 32 | >128 | >64 | 32 | >64 | 4 |
| HNCF44W | *Proteus cibarius* | Henan | >16 | 32 | >64 | >64 | >64 | >256 | 4 | 64 | >128 | >64 | 64 | >64 | 8 |
| CHNCF44W | *E.coli*, transconjugant | Henan | 16 | 2 | >64 | >64 | >64 | ≤0.125 | ≤0.125 | ≤0.5 | 32 | ≤0.5 | 16 | >64 | 2 |
| HNCF11W | *E. fergusonii* | Henan | ≤0.03 | 64 | >64 | >64 | >64 | ≤0.125 | ≤0.125 | 32 | >128 | >64 | 32 | 64 | 0.5 |
| CHNCF11W | *E.coli*, transconjugant | Henan | 0.06 | 32 | >64 | >64 | >64 | ≤0.125 | ≤0.125 | 16 | >128 | >64 | 32 | 4 | 1 |

^a^ MEM, meropenem; DOX, doxycycline; AMK, amikacin; FFC, florfenicol; KAN, kanamycin; CL, colistin; CIP, ciprofloxacin; TIG, tigecycline; OXY, oxytetracycline; TET, tetracycline; MIN minocycline; CFF, ceftiofur; ENR, enrofloxacin.

**Table S2. Antimicrobial susceptibility testing of the strains against tetracyclines.**

| IDs^a^ | Species | Antimicrobials (MICs, mg/L) | | | | |  | |  | |  |
| --- | --- | --- | --- | --- | --- | --- | --- | --- | --- | --- | --- |
|  |  | DOX^a^ | TIG | OXY | TET | MIN | | OMA | | ERA | |
| HNCF44W | *Proteus cibarius* | 64 | 32 | >128 | >64 | 32 | | 32 | | 64 | |
| DH5α-pMD19-T-tet(X6) | *E.coli* | 32 | 16 | 128 | 64 | 32 | | 16 | | 32 | |
| DH5α-pMD19-T | *E.coli* | 2 | 0.5 | 2 | 2 | 2 | | ≤0.125 | | 1 | |

^a^ DOX, doxycycline; TIG, tigecycline; OXY oxytetracycline; TET, tetracycline; MIN minocycline; OMA, omadacycline; ERA eravacycline.

**Supplementary Figure 1. S1-PFGE of three *tet*(X)-bearing strains and one transconjugant identified in this study.** M stands for the molecular marker derived from *Salmonella* serotype Braenderup strain H9812.


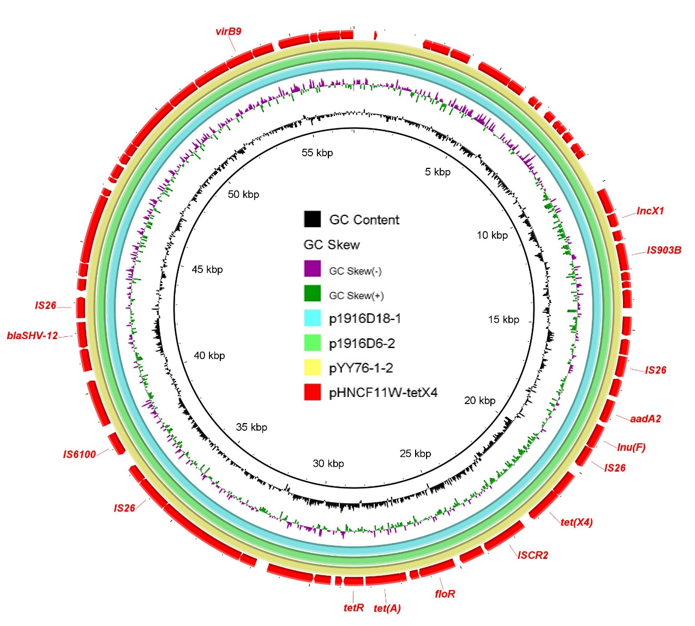


**Supplementary Figure 2. Alignment of plasmid pHNCF11W-tetX4 identified in this study with the other similar plasmids.** The outmost red circle denotes the plasmid pHNCF11W-tetX4. The gaps show regions that were missing in the respective plasmid compared with the reference plasmid.


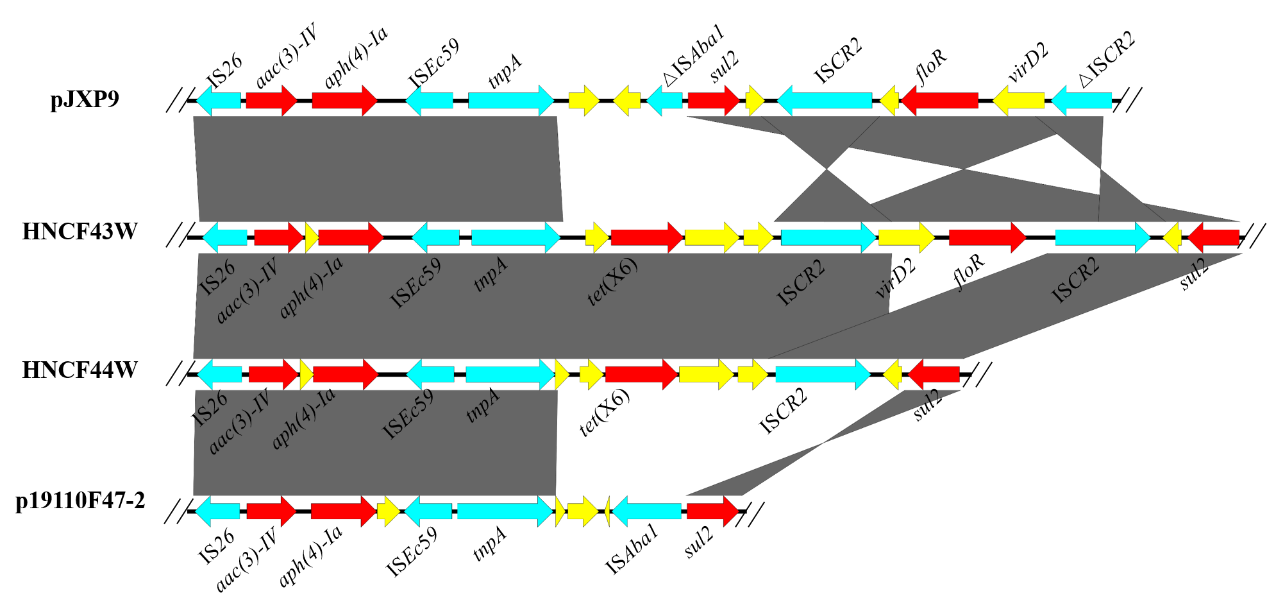


**Supplementary Figure 3. Comparative analysis of the *tet*(X6)-harbouring region and other similar structures.** The red arrows denote the resistance gene; the blue arrows denote the mobile element. The shadow parallelograms denote genetic regions that exhibit sequence homology among different segments.
